# Rapid spread of mouse mammary tumor virus in cultured human breast cells

**DOI:** 10.1186/1742-4690-4-73

**Published:** 2007-10-11

**Authors:** Stanislav Indik, Walter H Günzburg, Pavel Kulich, Brian Salmons, Francoise Rouault

**Affiliations:** 1 Research Institute for Virology and Biomedicine, University of Veterinary Medicine Vienna, Vienna, A-1210, Austria; 2 Christian-Doppler Laboratory for Gene Therapeutic Vector Development, Vienna, A-1210, Austria; 3 Veterinary Research Institute, Brno, 62100, Czech Republic; 4 Austrianova Biotechnology GmbH, Vienna, A-1210, Austria

## Abstract

**Background:**

The role of mouse mammary tumor virus (MMTV) as a causative agent in human breast carcinogenesis has recently been the subject of renewed interest. The proposed model is based on the detection of MMTV sequences in human breast cancer but not in healthy breast tissue. One of the main drawbacks to this model, however, was that until now human cells had not been demonstrated to sustain productive MMTV infection.

**Results:**

Here, we show for the first time the rapid spread of mouse mammary tumor virus, MMTV(GR), in cultured human mammary cells (Hs578T), ultimately leading to the infection of every cell in culture. The replication of the virus was monitored by quantitative PCR, quantitative RT-PCR and immunofluorescence imaging. The infected human cells expressed, upon cultivation with dexamethasone, MMTV structural proteins and released spiked B-type virions, the infectivity of which could be neutralized by anti-MMTV antibody. Replication of the virus was efficiently blocked by an inhibitor of reverse transcription, 3'-azido-3'-deoxythymidine. The human origin of the infected cells was confirmed by determining a number of integration sites hosting the provirus, which were unequivocally identified as human sequences.

**Conclusion:**

Taken together, our results show that human cells can support replication of mouse mammary tumor virus.

## Background

It is generally accepted that environmental factors play a role in the etiology of various types of cancer. This is most clearly demonstrated by epidemiological studies comparing the incidence of various cancers in migrating populations which tends to adopt the cancer incidence in the host country. However, despite tremendous efforts, the identification of such factors remains often elusive.

The involvement of mouse mammary tumor virus (MMTV), known to be associated with mammary carcinomas and T-cell lymphomas in mice, in human pathogenesis was based on immunological and molecular-biological evidence and proposed long ago (reviewed in [1]). The model became controversial due to the finding that the human genome carries endogenous sequences (HERV-K) displaying sequence similarity with MMTV, thereby making it difficult to distinguish the contribution of MMTV from that of HERV (reviewed in [2]). However, recently there has been renewed interest in this model due to the finding of Pogo's [3] and other groups [4-7], who identified MMTV sequences in human mammary carcinomas and primary biliary cirrhosis samples. Although it appears that the copy number of MMTV sequences in cancer samples is rather low, causing difficulties in their identification, the proviral sequences could be identified exclusively in transformed but not in non-malignant tissues [8]. Moreover, these sequences could be clearly distinguished from those present in the human genome, strongly indicating that they were acquired exogenously by infection [9].

However, although a growing body of evidence suggests a possible role for MMTV in human breast carcinogenesis [10] and possibly other human diseases such as primary biliary cirrhosis, the contribution of MMTV to the genesis of human tumors is still questioned. Beside the fact that some laboratories could not detect the MMTV sequences in human breast tumors [2,11], this skepticism is largely due to a deep-seated dogma that MMTV is exclusively a mouse virus, unable to infect human cells and hence without the capacity to trigger any human illness.

Contrary to this traditional view we could recently demonstrate that both a wild-type and a genetically modified virus carrying EGFP (MMTV-EGFP) can infect a number of different cultured human cells [12]. Moreover, the infectious titer obtained on human cells was similar to the titer obtained on cultured mouse mammary tumor cells (NMuMG). Importantly, the infection was neutralized by specific anti-MMTV serum and mutation of the *env *gene in the molecular clone completely abrogated infection, providing evidence for specific, infection-mediated transfer of MMTV to the target human cells [12]. Nevertheless, although authentic infection of human cells was demonstrated, the ability of MMTV to productively replicate in human cells was not addressed by these studies.

Here we demonstrate that MMTV rapidly spreads in cultured human breast cells, ultimately leading to the infection of all the cells in culture, thus providing further evidence that human cells are compatible hosts for MMTV. Our observations further suggest that cross-species transmission of MMTV is in general possible and strengthens the contention that MMTV might be an etiological agent involved in human breast carcinogenesis.

## Results

### Infection of Hs578T cells

Previously we have shown that wild type, MMTV(GR), and genetically marked MMTV-EGFP virus, could infect cultured human cells via a specific interaction of the viral envelope with the cell surface receptor [12]. Here we have extended this earlier work and addressed the question of whether MMTV can productively infect human cells. To assess the ability of the wild type virus, MMTV(GR), to infect and spread in the human breast carcinoma cell line, Hs578T, we transduced the cells with cell-free virus taken from supernatants of GR cells, a mouse mammary tumor derived cell line that produces MMTV [13]. Simultaneously, the identical virus was used to infect feline kidney cells, CrFK, that are known to support replication of MMTV[14]. Subsequently, the presence of MMTV in the infected cells was monitored by a PCR assay which targeted a 717 bp segment of the LTR-*gag *region and allowed detection of the provirus in DNA of the infected cells.

**Figure 1:**
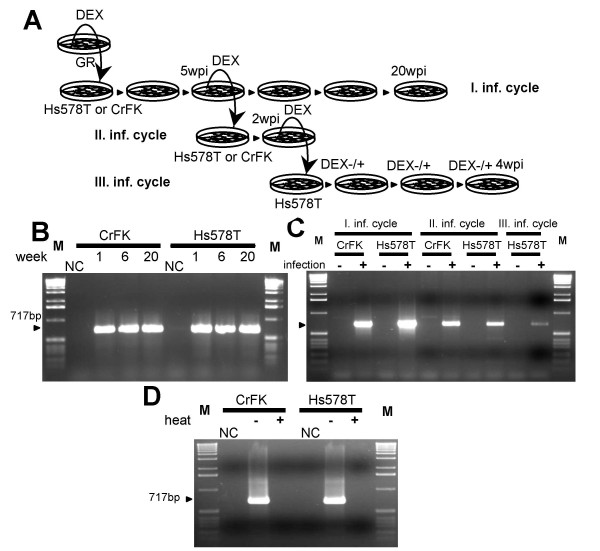
**Infection of human breast cell line Hs578T and feline kidney cells, CrFK, with MMTV(GR) virus**. (A) Experimental design. Wpi: weeks post infection. (B) The cells infected with MMTV(GR) were monitored for 20 weeks. Genomic DNA was harvested at week one, six and 20 after infection, respectively, and analyzed by PCR for the presence of MMTV sequences. NC: non-infected cells. M: 1 kb marker. (C) Three infectious cycles were performed in Hs578T cells. The cells infected with MMTV(GR) virus are denoted as first infection cycle. The cell culture supernatant from these Hs578T cells was used in a subsequent infection round. Medium from the second-cycle infected Hs578T cells was used for third infection cycle. M: 1 kb marker. (D) Heat inactivation of the MMTV(GR). Where indicated (heat +) was the virus subjected to the heat treatment (60°C for 10 min). NC: non-infected cells, M: 1 kb marker.

Both human (Hs578T) and feline (CrFK), cell lines became infected since the MMTV proviral fragment was readily detectable in the PCR assay (Figure 1B). Importantly, heat inactivation of the virus abrogated the infection as no PCR products were detected in both cell types infected with the heat-treated virus (Figure 1D). The infected cells were cultured for five months and proviral DNA could be detected in both cell types throughout the whole duration of the experiment (Figure 1B), demonstrating that the cells became persistently infected. The primer pair used for PCR assay was specific for MMTV sequences since neither human nor feline endogenous retroviral sequences were amplified in reactions performed with uninfected cells (Figure 1B).

To ascertain whether the persistently infected human cells (five weeks after infection) produce infectious MMTV particles, the clarified and 0.45 μm filtered cell culture supernatant, collected from infected Hs578T cells 24 h after stimulation with 10^-6 ^M dexamethasone (DEX), was plated on fresh, uninfected, Hs578T or CrFK cells. One week after infection, the DNA from these cells was subjected to the MMTV-specific PCR analysis. A PCR product of the expected size of 717 bp was obtained from the DNA of infected cells, but not from that of mock-infected cells (Figure 1C). Despite the decreased intensity of the PCR signal in the second round of infection as compared to the original infection with MMTV(GR) virus, this result clearly indicates that infected human cells release virions capable of infecting cells in culture.

To extend this analysis, an additional round of infection was also performed. First, the expression of MMTV in the second-round infected human cells was induced by 10^-6 ^M DEX treatment and the cell-free supernatant was used for another round of infection. As in the previous case, the analysis of the target cell DNA by PCR was performed seven days after infection and revealed a weak but detectable PCR product, indicating another successful infection cycle in human cells (Figure 1C, only human cells are shown).

**Figure 2:**
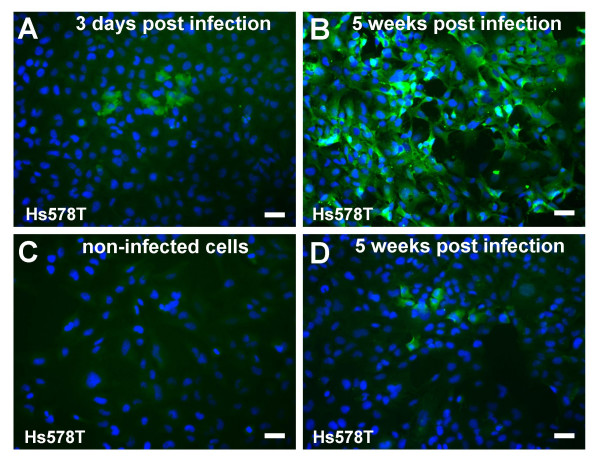
**Immunofluorescence imaging of MMTV-infected Hs578T cells**. The expression of capsid proteins in the third-round infected Hs578T cells was visualized by immunofluorescence staining using a monospecific anti-CA serum. Only a small number of MMTV-positive cells was detected in the third-round infected cells 3 days after infection (A), whereas by week five all the cells expressed MMTV antigen (B). The increase was strictly DEX dependent. Upon cultivation of the cells in DEX-free medium no increase in the number of CA-positive cells could be observed (D). (C) Non-infected Hs578T cells. 24 h prior immunostaining all the cells (A, B, C, D) were grown in medium supplemented with 10^-6 ^M DEX. (Scale bar, 50 μm).

To demonstrate the production of MMTV-specific structural proteins and further confirm the PCR results, an indirect immunofluorescence staining with mono-specific anti-MMTV-CA polyclonal serum was performed. Consistent with the DNA analysis, the expression of the core protein was detected in the third-round infected cells (Figure 2A). The antigen-expressing cells were grouped in small clusters. These clusters appear to result from division of a single infected cell during the 72-h incubation period or/and possibly by cell-to-cell spread of the virus. The absence of the fluorescence signal in non-infected cells shows the specificity of the reaction (Figure 2C).

**Figure 3:**
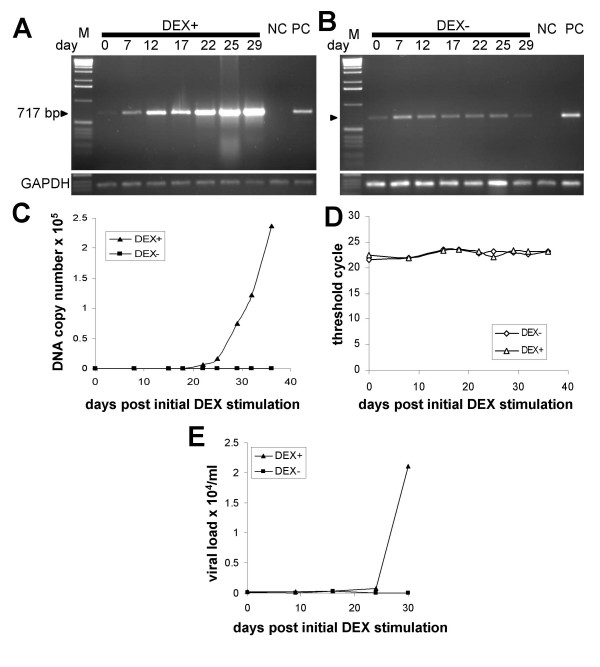
**Quantification of proviral DNA and viral RNA in cell lysates and supernatants of the third-round infected human breast cells during a time-course experiment**. (A and B) The third-round infected cells were cultured in the presence (A) or absence (B) of 10^-6 ^M DEX. Genomic DNA was extracted from the infected cells at the indicated time points and semiquantitative PCR was performed. NC: non-transduced HS578T cells. PC: second-round infected Hs578T cells. Equal DNA loading was controlled in a PCR assay with GAPDH-specific primers (bottom panels). M: 1 kb marker. (C) Real-time TaqMan PCR quantifying proviral loads in the infected Hs578T cells during the time-course experiment. (D) Equal loading of the PCR reactions was controlled in a Real-time TaqMan PCR specific for GAPDH gene. (E) The viral RNA was quantified by Real-time RT-PCR in cell culture fluids of the infected Hs578T cells grown either in the presence or absence of 10^-6 ^M DEX.

In subsequent time-course experiments we sought to directly demonstrate the productive replication of MMTV in human breast cells. We reasoned that if human cells are capable of supporting replication of MMTV, then we should observe increasing levels of proviral DNA upon cultivation of the third-round infected cells in the presence of 10^-6 ^M DEX, a glucocorticoid inducing MMTV expression. Indeed, as expected for an ongoing productive infection, stronger PCR signals were detected at later cultivation time points (Figure 3A). The increased intensities of the PCR signals could not be attributable to unequal loading of the amplification reaction, since a PCR assay with GAPDH-specific primers performed with the identical template amounts, showed similar levels of PCR products at all time points (Figure 3A and 3B, bottom). Furthermore, no increase in the intensity of the PCR products were detected in the cells cultured without DEX (Figure 3B). Taking this data together, we observed a time- and dexamethasone-dependent increase of MMTV-specific PCR products strongly supporting the productive infection of the human cells with MMTV. Similar results were also observed when a TaqMan Real-time PCR assay targeting the 5' end of the *env *coding region was used for an accurate quantification of the proviral load in the infected cells during the time-course experiment (Figure 3C). Real-time PCR assays of the GAPDH housekeeping gene confirmed equal loading of all PCR reactions (Figure 3D).

Replication of the virus in human cells was further demonstrated by quantification of viral RNA load in the culture supernatant of the third-round infected cells. As expected for ongoing replication of MMTV in human cells, substantially more viral RNA was detected in the medium of DEX-induced but not in that of non-induced cells at later cultivation time points (Figure 3E).

### All the cells cultured in the presence of DEX were infected

**Figure 4:**
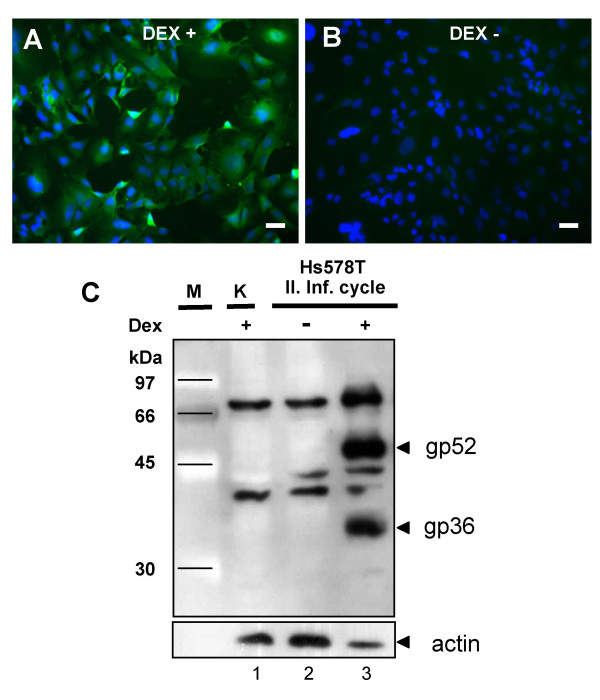
**Detection of expression of MMTV proteins in the infected human cells**. (A and B) Infected Hs578T cells were cultured for 5 weeks in the presence of 10^-6 ^M DEX. One week before immunofluorescence staining the cells were cultivated in the absence of the glucocorticoid analog and 24 h prior immunofluorescence staining with anti-CA antibodies the production of MMTV-specific proteins was either induced (A) or not (B) by addition of 10^-6 ^M DEX in the cell culture media. The nuclei of the cells were counterstained with DAPI. (C) Western blot detecting the expression of gp52 and gp36 Env proteins in the second-round infected HS578T cells. Lane 1, non-infected Hs578T cells, NC; lane 2, infected human cells not stimulated with DEX; lane 3, infected human cells in which the expression of MMTV structural proteins was induced by 10^-6 ^M DEX 24 h before protein harvest.

To further demonstrate the replication of MMTV in the human cells exposed to MMTV(GR) virus, indirect immunofluorescence imaging of the MMTV core was performed. A marked difference in the numbers of MMTV core protein expressing cells was detected in a time-course experiment. Whereas only a small number of MMTV-positive cells were detected in the third-round infected cells shortly after infection (Figure 2A), by week five all the cells expressed MMTV antigen (Figure 2B, Figure 4A). The increase was strictly DEX dependent. Upon cultivation of the cells in DEX-free medium no increase in the number of CA-positive cells could be observed (Figure 2D).

Moreover, as expected for the expression of proteins driven by the MMTV LTR promoter, the production of the core proteins could be found only in the cells when DEX was added 24 h prior to immunostaining (Figure 4A). Similarly, Western blot analysis conducted with anti-gp52/36 antibody revealed the presence of MMTV Env proteins, gp52 and gp36, only in the infected cells, in which the production of proteins was induced by DEX. Neither gp52 nor gp36 could be visualized from protein extracts of cells kept under DEX-free conditions or uninfected cells that were cultured in medium containing 10^-6 ^M DEX 24 h before the immunostaining (Figure 4C).

### Infection was neutralized by anti-MMTV serum and abolished by AZT

**Figure 5:**
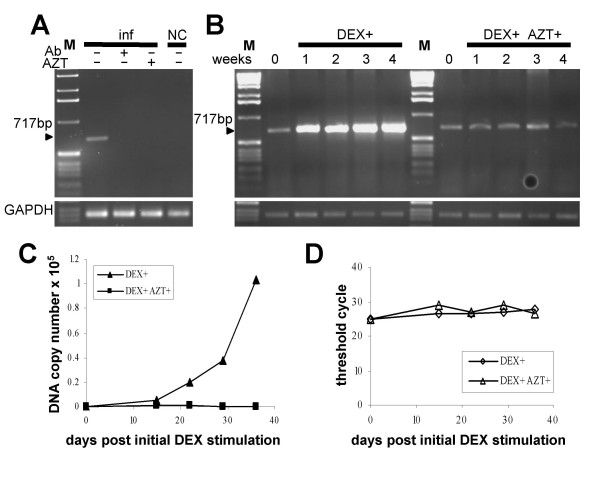
**Neutralization of viral infectivity and AZT treatment**. (A) The presence of proviral DNA in the infected Hs578T cells was determined by PCR. The virus released from the second round infected Hs578T cells was, prior infection, pre-incubated either with anti-MMTV neutralizing antibody (Ab) or PBS. Where indicated AZT was added to the cells infected with the virus. NC: non-infected Hs578T cells. M: 1 kb marker. (B) Spread of the virus was abrogated in medium containing AZT. The third-round infected Hs578T cells were cultured for four weeks in medium containing DEX either supplemented with AZT or not and the presence proviral DNA was monitored by a semiquantitative PCR. GAPDH-specific PCR was used to demonstrate equal loading of all PCR reactions (bottom panels). M: 1 kb marker. (C) Real-time TaqMan PCR quantifying proviral loads in the infected Hs578T cells during the AZT treatment experiment. (D) Equal loading was contolled in a Real-time TaqMan PCR specific for GAPDH gene.

To characterize the infectious particles produced from the infected human cells, we carried out a neutralization assay using anti-MMTV serum. As shown in Figure 5A, the serum blocked the infectivity of the virus, as no MMTV specific signal could be detected in PCR analysis of the cells exposed to virus-anti-MMTV serum mixture. The specific neutralization of infectivity confirms that the infectious particles produced by the infected Hs578T cells are antigenically related to the virus produced in murine cells.

Reverse transcription (RT) is an obligatory step in the replication cycle of retroviruses, hence by blocking RT activity viral infection can be abolished. We used 3'-azido-2'-deoxythymidine (AZT), a potent inhibitor of RT activity, to inhibit the infection of human cells by MMTV. The addition of AZT to the culture medium of the infected cells rendered the virus unable to undergo RT and again, no MMTV specific signal could be detected in PCR analysis of DNA from the infected cells (Figure 5A). These results, in conjunction with the neutralization of viral infectivity with specific serum, confirm that the observed results are due to authentic infection rather than an artifact e.g. due to the carry-over of proviral DNA from virus producing cells.

Having validated the inhibitory potential of AZT in a single-round infection experiment, we sought to determine whether AZT can also inhibit spread of the virus in cultures of infected human cells. In a time-course experiment carried out in a similar manner as outlined previously, the third-round infected Hs578T cells were cultured in 10^-6 ^M DEX-containing medium either in the absence or presence of AZT. Whereas MMTV spread, as indicated by the increasing PCR signal over time, was readily observed in the cells growing in medium without AZT, no increase in the MMTV-specific PCR signal was seen in AZT treated cells (Figure 5B, Figure 5C). Thus, AZT at a concentration of 10 μM, inhibited the spread of the virus in human cells without having a significant effect on the morphology or the rate of growth of the cells (data not shown).

### Visualization of viral particles released from infected human cells

**Figure 6:**
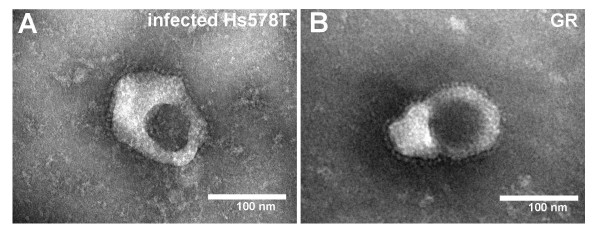
Electron microscopy of viral particles released from the infected Hs578T (A) and GR cells (B).

The production of MMTV particles by the third-round infected Hs578T cells was confirmed by electron microscopy. Cells cultured for five weeks in medium containing 10^-6 ^M DEX and expressing MMTV-specific antigen as shown by immunostaining, were used as a source of the virus for electron microscopy. Negatively stained, high-speed centrifugation pellets contained particles morphologically resembling those of MMTV (Figure 6A and 6B). Characteristic prominent glycoprotein knobs on the surface of the virions, a hallmark of MMTV, were clearly visible. The eccentrically placed nucleoid, another characteristic of B-type viruses as well as the expected diameter of the particles (~130 nm), together with the fact that no comparable virus-like particles were found in non-infected cell supernatants, provided further evidence that the observed structure is MMTV. The presence of spiked virions in culture medium several months after the initial inoculation of the cells with MMTV(GR) virus is unlikely to be due to carry-over of residual virus but rather is indicative of virus replication in human cells.

### Determination of integration sites and host-sequence duplications flanking proviruses

**Figure 7:**
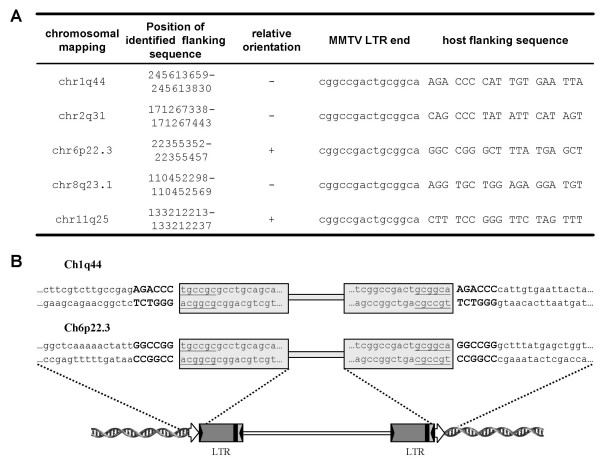
**Virus-host junction sequences**. (A) The junctions detected by LM-PCR in Hs578T cells infected with MMTV(GR). Terminal sequence of MMTV LTR (small letters) and 18 nucleotides of host flanking sequence (capital letters) are shown. Determined host sequence was mapped using a BLAT search at the UCSC Genome Bioinformatics group web page. The exact position of the host sequence amplified in LM-PCR on the chromosome is numbered according to Human Mar. 2006 (hg18) assembly. (B) Duplications of 6 bp long host provirus flanking sequences were determined. MMTV proviral sequences are boxed, inverted repeats at the end of LTRs are underlined. Duplications of host flanking sequences are indicated by large bold letters. Schematic diagram of an integrated MMTV provirus is shown below. Direct repeats of the host sequence are indicated by open arrows. Inverted repeats terminating the LTR are shown as inverted solid triangles.

An essential step in the retroviral replication cycle is the integration of the provirus into the host genome. To investigate whether the MMTV(GR) proviral DNA is inserted in the genome of the infected human cells, thereby providing conclusive evidence of infection of human cells, we performed LM-PCR as described previously by Wu et al. [15]. Genomic DNA harvested from human and feline cells transduced in the second infection cycle was used for digestion with MseI, ligation with linker and subsequent amplification of virus-host junction sequences. A number of LM-PCR products were readily detected in both human and feline infected cell lines. Of these, five integration sites determined in the infected Hs578T cells were characterized further. All five human integrants could be unambiguously mapped to a locus in the human genome (Figure 7A).

The integration process, performed by the virally encoded integrase, is a remarkably accurate process displaying striking similarities with that of other transposable elements. A hallmark of the retroviral integration process is the duplication of a short cellular sequence of four to six bp, at the integration site, with a 6 bp duplication being characteristic of the MMTV integration process [16]. Importantly, as we determined the 6 bp-long duplications flanking the provirus we concluded that the human cells acquired the MMTV(GR) proviral DNA via a legitimate infection rather then by other non-specific means (Figure 7C).

### Virus infecting human cells is not a recombinant virus

**Figure 8:**
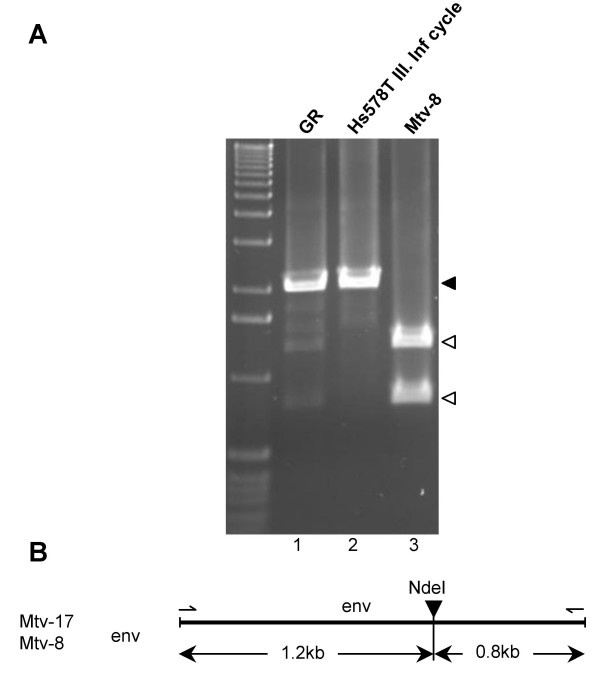
**Virus infecting human cells do not contain *env *gene derived from endogenous retroviruses**. (A) The PCR products encompassing complete *env *coding region amplified from the cell lysates of GR cells (lane 1) and third-round infected Hs578T cells (lane 2) were submitted to digestion with NdeI. As a digestion control, PCR product obtained by amplification of MTV-8 *env *sequences using pGR16 plasmid as a template, was digested with the same restriction enzyme (lane 3); M, 1 kb marker; black arrow indicates undigested product; open arrows denote fragments resulting from NdeI digestion. (B) Schematic drawing showing NdeI site in the *env *gene of the Mtv-17 and Mtv-8 viruses and the length of the respective restriction fragments.

It was previously reported that endogenous MMTVs (Mtvs), which are expressed in cells infected by exogenous virus, could co-package and recombine with exogenous viruses. Tumors that have arisen in GR mice as a result of infection of mammary gland cells were reported to carry recombinant proviruses in which the *gag-pol *region was derived from the Mtv-2 virus and the *env *gene was derived from the Mtv-17 endogenous virus [17,18]. Exchange of the *env *coding sequence could, in turn, result in an altered virus tropism. Since, in the initial infection of human cells, we used virus obtained from the GR tumor derived cell line, we sought to determine whether such a recombination, altering virus tropism, and possibly enabling infection of human cells, had occurred. We examined the newly integrated proviruses in the infected human cells for the presence of a recombinant virus carrying the *env *gene (or its part) derived from endogenous MMTVs or possibly from other endogenous retroviral sequences closely related to MMTV. We reasoned that if the infection of human cells is mediated by an envelope protein encoded by a provirus other than Mtv-2 and this, in turn, allows the infection of human cells, then such *env *gene sequences should be enriched in proviruses passaged in human cells. Hence, we used the third-round infected Hs578T cells, in which MMTV has undergone several rounds of replication (the cells were cultured in DEX-containing medium for five weeks) as a source of proviral DNA. The *env *gene was amplified, cloned into a vector and sequenced. Alternatively, the PCR product was digested with NdeI, an enzyme which cuts *env *sequences of the endogenous Mtvs present in GR cells (Mtv-17, Mtv-8) but does not cleave within the Mtv-2 *env *gene. The *env *coding region amplified from the infected human cells was not digested, suggesting that endogenous Mtv *env *(Mtv-8 and Mtv-17) sequences are not present in the infected cells (Figure 8).

**Figure 9:**
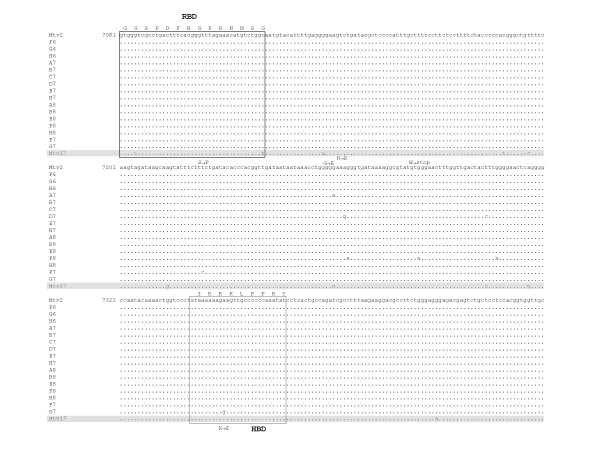
**Alignment of a partial *env *gene sequences from the third infection cycle of Hs578T cells**. Genomic DNA extracted from the third-round infected Hs578T cells, was used for amplification and cloning of MMTV *env *sequences. Sequences of sixteen clones were aligned. Mtv-2 and Mtv-17 (accession number AF263910, sequence is shaded) sequences were included in the alignment. Putative heparin binding domain (HBD) and receptor binding site (RBS) are boxed and amino acid residues representing these regions are shown above the boxes. Non-synonymous mutations in proviral sequences from infected cells resulting in an amino acid exchange are indicated. The coordinates of the Mtv2 nucleotide sequence are according to the MMTV reference strain (accession number M15122).

The sequences obtained from sixteen different plasmid clones were aligned and compared with the Mtv-2 as well as Mtv-17 *env *coding regions (Figure 9). Although several point mutations were detected, all the sequences could be identified as Mtv-2 sequences. Interestingly, most of the mutations were found between the putative receptor binding site (RBS) and heparin binding domains (HBD), the regions that are thought to play a role in the interaction with the receptor and hence are most likely located at the outer part of the protein [19]. Additionally, one non-synonymous mutation leading to a transition from the basic amino acid lysine to the acidic glutamic acid was identified in the putative HBD.

To find out if recombination took place in other regions of viral genome, complete proviral DNA was amplified and cloned into the pCR-XL-TOPO vector. Sequence analysis of two proviral genomes did not reveal any recombination events either with endogenous MMTVs or with human endogenous retroviral sequences. The sequences, beside some point mutations, which most likely arose during error-prone reverse transcription, matched the original Mtv-2 sequences (data not shown). Taken together, this strongly suggests that the Mtv-2 virus *per se *is responsible for the observed virus spread and thus has the capacity to replicate in human cells.

## Discussion

The achilles heel to the model linking MMTV infection with human breast cancer, which was recently revitalized after detection of MMTV sequences in human cancers [3-8], is the widely believed dogma that human cells are not a compatible host for MMTV. Contrary to this traditional view, we have previously shown that wild type MMTV(GR), and genetically marked MMTV-EGFP virus infects human cells [12]. Here we provide further evidence, including increasing levels of proviral DNA within infected cells over time, as well as viral RNA in the supernatant of infected cells and specific immunofluorescence imaging, strongly arguing that wild type MMTV has the potential to replicate in human cells.

The infected cells were unambiguously of human origin and no accidental contamination by mouse or other cells occurred. All five identified integration sites hosting MMTV provirus were unequivocally identified as human sequences and could be mapped to loci in the human genome (Figure 7A). Moreover, the canonical termination of proviral sequences (CA dinucleotide), 6 bp-long duplications of host sequences flanking the provirus, together with the fact that the virus supernatant used for infections was filtered (0.45 μm) argues that the cells acquired MMTV by a genuine infection and not by other non-specific means.

Importantly, the virus replicating in human cells was not a recombinant virus as could be confirmed by the analysis of two complete proviral sequences recovered from the third-round infected Hs578T cells. This was further supported by restriction digestion analysis (Figure 8) as well as sequencing analyses of independent *env *gene sequences from infected human cells (Figure 9). Taken together, we did not observe any recombination event, neither with MMTV nor with other human endogenous sequences that would predispose the MMTV virus for replication in human cells. These results, in conjunction with the neutralization of infectivity of the original MMTV(GR) as well as of the virus released from infected human cells with the specific anti-MMTV serum (Figure 5A) and our previous observation that mutation of *env *leads to complete abrogation of viral infectivity [12], provide strong support for the utilization of the Mtv-2 Env as the human cell entry ligand.

Notably, we did not detect any mutation in the putative RBS of the Env protein. Interestingly, a number of non-synonymous mutations were located between the putative RBS and HBD, the regions previously reported to be involved in the interaction with the cell membrane. The identification of the RBS as well as of the HBD, was based on a structural alignment of MMTV surface Env protein with that of Friend murine leukemia virus (Fr-MLV), for which the crystal structure and functional domains had been previously solved [20,19]. Notably, although the putative receptor binding site of MMTV Env is predicted to be located at the external part of the polypeptide, the location of the domain does not match the position of the receptor binding domains of Fr-MLV Env. Interestingly, the sequence divergent region between RBS and HBD, resembles the variable region A (VRA) of Fr-MLV Env, which is known to be critical for receptor interaction [21,22]. In this light it is then conceivable that this region is also involved in virus-cell interaction and that mutations at such positions could alter virus tropism and possibly predispose MMTV for infection of human cells. This concept is also supported by the fact that unique amino acid residue changes, relative to all known exogenous and endogenous MMTVs, were identified in this region from several MMTV-like elements isolated from primary human breast cancer samples [19]. Although, the alteration in this region was not found in all the sixteen *env *clones amplified from the infected human cells and hence it appears that it is not mandatory for human cell infections, the high number of non-silent mutations suggests that this region is under selection, in the virus passaged in human cells.

A single non-synonymous mutation was also detected in the putative heparin binding domain. Cell surface glycosaminoglycans, in particular heparan sulfate, have been proposed to mediate the attachment of a variety of viruses e.g. human immunodeficiency virus type 1 (HIV-1) to target cells, thus increasing the chance of receptor binding [23]. Since the net charge of the HBD seems to be important for the interaction of this domain with negatively charged cellular polymers, replacement of basic lysine amino acid residues by acidic glutamic acid could alter the affinity of the virus for the cell surface. Nonetheless, such mutation was detected only in one of sixteen clones hence its significance remains questionable.

## Conclusion

Despite the widely accepted belief that human cells are not appropriate host for MMTV, our data demonstrate the productive infection of human breast cells. This finding might help to explain the presence of MMTV-like sequences in at least a certain proportion of human breast cancers and primary biliary cirrhosis patients. Although our study does not prove causality, it certainly lends more weight to the hypothesis linking MMTV and human disease and might further substantiate the notion that MMTV may be involved in human diseases such as breast cancer and primary biliary cirrhosis.

## Methods

### Cell culture and infection

GR cells, a well characterized cell line established from an MMTV-induced mouse adenocarcinoma [13], were used for virus production as described previously [12]. The human mammary carcinoma, Hs578T [24], and feline kidney, CrFK [14], cell lines cultured in DMEM supplemented with 10% FCS were seeded, 24 h prior infection, in six-well plates at a concentration of 2 × 10^4 ^cells per well. The filtered (0.45 μm, Sarstedt) virus supernatant containing polybrene (8 μg/ml) was incubated with the target cells for 2 hours and fresh medium was then added to the cells. The infected cells were cultured for 20 weeks and the presence of MMTV proviral sequences was monitored by PCR. Five weeks after the initial infection, the human cells were stimulated with 10^-6 ^M dexamethasone (DEX) and, 24 h after induction, the filtered cell culture supernatant was used for the infection of either Hs578T cells or CrFK cells. These second-round infected Hs578T cells were cultured for two weeks and, after induction with DEX, used as a source of MMTV for the third-round infection of Hs578T cells. The human cells infected in the third-round were further cultured in DEX-containing medium (10^-6^M) for five weeks and viral spread was monitored as described below (Figure 1A).

### Immunofluorescence imaging

The expression of MMTV structural proteins in 10^-6 ^M DEX induced infected human cells was determined by indirect immunofluorescence staining. A monospecific serum (dilution 1:200; kindly provided by Michael Sakalian) raised in rabbits immunized with an E. coli expressed His-tagged MMTV CA protein, was used together with a FITC-conjugated anti-rabbit IgG (DAKO) diluted 1:30 for the imaging. The nuclei were subsequently counterstained with DAPI (Roche) and the slides were examined with a Zeiss Axiovert 200 M microscope.

### Western blot

The cellular extracts of the infected human cells, with or without stimulation with 10^-6 ^M DEX 24 h before the protein harvest, were separated by electophoresis on a 10% polyacrylamide gel, transferred to a PVDF membrane and the membrane was allowed to react with an anti-gp52/36 antibody at a dilution of 1: 12,000 (a generous gift from Janet Butel). After incubation with horseradish peroxidase-linked anti-rabbit IgG antibody (DAKO, dilution 1: 10,000), the Env proteins were revealed using an ECL Plus kit (Amersham).

### PCR

The presence of MMTV(GR) proviral DNA in infected cells was determined as described previously [12]. 300 ng of genomic DNA was used as a template for the amplification. Equal loading of each PCR reaction was controlled using a GAPDH-specific primer pair (GAPDH F: 5'ATGGCTCCTGCACCACCAAC 3'; GAPDH R: 5' CGCCTGCTTCACCACCTTCT 3') in a PCR reaction (only 25 cycles) carried out with the identical sample amounts as in the MMTV-specific PCR.

### Real-time TaqMan PCR

The proviral loads in the infected cells were quantified by a Real-time TaqMan PCR using the following set of primers: MMTV 01F: 5' GGAAAGTCCGGAGGATGAATCTA 3', MMTV 02R: 5' CTCCGCTTCGGAGATTAACG 3'and 6FAM/TAMRA TaqMan probe: 5' CATCAAAGAGAAGACGGCTTGGCAACATC 3'using 50 ng of genomic DNA as a template. TaqMan reactions were carried out in a total volume of 25 μl containing 1 unit BioTaq (Q Biogene), 1× PCR buffer, 3 mM MgCl_2_, 200 μM dNTPs, 300 nM forward primer, 300 nM reverse primer and 200 nM TaqMan probe. In each TaqMan experiment, a standard was run consisting of a serially diluted plasmid carrying a molecular clone of MMTV (pGR102) [25]. An Mx3000P QPCR System (Stratagene) was used with the following thermal cycling program (95°C for 2 min followed by 40 cycles of 95°C for 30 s and 60°C for 1 min). The threshold cycle (*C*_τ_) was measured for each well and a standard curve was plotted using the threshold cycle values of the serially diluted plasmid DNA. Equal loading of PCR reactions was verified using a TaqMan Real-time PCR specific for GAPDH gene using following primers and FAM/TAMRA TaqMan probe: GAPDH F: 5'ATT CCA CCC ATG GCA AAT TC 3', GAPDH R: 5'CGC TCC TGG AAA TGG TGA T 3', GAPDH P: 5'TGG CAC CGT CAA GGC TGA GAA CG 3'. Identical PCR conditions as outlined above were employed.

### TaqMan RT-PCR

RNA was isolated using the QIAamp viral RNA kit (Qiagen), treated with DNaseI, and reverse transcribed in 20 μl using an MMTV-specific primer (8649-: 5'GTGTAGGACACTCTCGGGAGTTC 3') and Superscript II reverse transcriptase (Invitrogen). 8 μl of the RT reaction was subsequently used in the Real-time TaqMan quantitative PCR as described above. An RNA standard was prepared by *in vitro *transcription of pCMVenv plasmid [26] that had been linearized by XbaI digestion, using T7 RNA polymerase (Invitrogene). Prior to use in the quantitative PCR, the RNA was treated with DNAse I and reverse transcribed as outlined above.

### Neutralization of viral infectivity, heat inactivation and AZT treatment

Neutralization of viral infectivity and heat inactivation was performed as described previously [12]. The anti-MMTV potency of 3'-azido-3'-deoxythymidine (AZT) (Sigma) was first investigated in a single round infection experiment. 10 μM AZT was added simultaneously with the virus inoculum to the cells. After 2 h incubation at 37°C, the supernatant was replaced with fresh culture medium containing 10 μM AZT. The infection of cells was evaluated by PCR one week after infection. The virus spread experiment was performed with the third-cycle infected Hs578T cells. The cells were cultured in the cell culture medium supplemented with DEX (10^-6 ^M) either in the presence or absence of AZT (10 μM).

### Electron microscopy

The third-round infected Hs578T cells cultured for five weeks in the medium supplemented with 10^-6 ^M DEX were grown to confluence. The cell supernatant was clarified by a 15 min centrifugation at 1500 × g and ultracentrifuged for 2 h through a 20% sucrose cushion at 120,000 × g. The pellet was resuspended in TN buffer, negatively stained with ammonium molybdate acetate and observed in a Philips 208 S Morgagni transmission electron microscope.

### Integration site detection

Integration sites were determined as described previously [15]. Briefly, the LM-PCR was performed with one primer specific to the linker [15] and other primer to the MMTV LTR (1370F: 5' CGTCTCCGCTCGTCACTTAT 3'). A semi-nested PCR was carried out using primers linker 2 [15] and 1370F. The PCR products were TA-cloned into pCR2.1 vector (Invitrogen) and sequenced. The obtained sequences contained the 3'LTR sequence from the 1370F primer to the end of 3'LTR, the linker sequence and unknown virus flanking sequences, which were identified using a BLAT Genome Browser (UCSC Genome Bioinformatics).

### Determination of six nt duplications

The sequences extending leftwards from the integrated proviruses were determined in a PCR using a MMTV-specific reverse primer 9400- (5' ACACCAAGGAGGTCTAGCTCTG 3') in combination with a human genome specific primer designed about 400 bp apart from the locus hosting MMTV provirus, which was determined by LM-PCR (chr1q44: 5'CACTGCCAATGCCTCCTTCC 3'; chr6p22.3: 5' GAGACAACCAAGGCAGTGAG 3'). The PCR was carried out using 500 ng of genomic DNA from the second-round infected Hs578T cells. The PCR products of expected size were excised from the agarose gel, purified and directly sequenced.

### NdeI digestion

The *env *gene from the infected human cells (five weeks in DEX-containing medium), GR cells and pGR16 plasmid carrying Mtv-8 provirus [25] was amplified as described above. The PCR products were purified and digested with NdeI (Promega). The resulting fragments were resolved on a 1.5% agarose gel and visualized by ethidium bromide staining.

### Env amplification and sequencing

The complete *env *coding segment was amplified with a primer pair 6684F (5'ATGCCGAAACACCAATCTG 3') and 8649-, using genomic DNA (300 ng) from the third-round infected human cells (cultured five weeks with 10^-6 ^DEX) as a template for PCR. The expand high fidelity system (Roche) was used according to the manufacturer' s instructions to minimize the introduction of mutations during PCR. The PCR products were cloned into the pCR2.1 vector and sequenced. The obtained sequences were aligned using the Align Plus 4 algorithm (Sci Ed Central).

### Complete provirus amplification and sequencing

500 ng of genomic DNA harvested from the third-round infected human cells (cultured for five weeks in 10^-6 ^M DEX containing medium), were used for a long-template PCR as recommended in Expand long template PCR system (Roche) using primers 1370F and 9877R (5' TCAGCACTCTTTTATATTATGG 3'. The PCR product was cloned into a pCR-XL-TOPO vector (Invitrogen) and sequenced using primers available from the authors on request.

## Competing interests

The author(s) declare that they have no competing interests.

## Authors' contributions

SI participated in the design of the study, carried out all the experiments and drafted the manuscript. PK performed electron microscopy, WHG, BS, FR participated in the design of the study and helped to draft the manuscript.
